# Diurnal Variation of L-Arginine and the Cardiovascular Risk Markers Asymmetric and Symmetric Dimethylarginine and Homoarginine in Rotating Night Shift Workers and Controls

**DOI:** 10.3390/biom13091282

**Published:** 2023-08-22

**Authors:** Juliane Hannemann, Debra J. Skene, Benita Middleton, Edzard Schwedhelm, Anika Laing, Rainer Böger

**Affiliations:** 1 Institute of Clinical Pharmacology and Toxicology, University Medical Center Hamburg-Eppendorf, 20246 Hamburg, Germany; ju.hannemann@uke.de (J.H.); schwedhelm@uke.de (E.S.); anika.laing@gmail.com (A.L.); 2 Chronobiology, Faculty of Health and Medical Sciences, University of Surrey, Guildford GU2 7XH, UK; d.skene@surrey.ac.uk (D.J.S.); b.middleton@surrey.ac.uk (B.M.)

**Keywords:** nitric oxide, L-arginine metabolism, asymmetric dimethylarginine, diurnal rhythm, rotating night shift work, blood pressure, melatonin, cortisol

## Abstract

Asymmetric dimethylarginine (ADMA) and symmetric dimethylarginine (SDMA) interfere with nitric oxide (NO) formation from L-arginine via different mechanisms. ADMA is a biomarker of cardiovascular disease and mortality, whilst SDMA is a biomarker of mortality after ischemic stroke. Homoarginine, another L-arginine-derived amino acid, is associated with stroke and congestive heart failure. Acute ischemic events like myocardial infarction show a time-of-day variation in the timing of their onset, as do NO-mediated vascular function and blood pressure. We studied whether the plasma concentrations of L-arginine-related amino acid metabolites show diurnal variation in a clinical study comparing 12 non-night shift workers with 60 rotating night shift workers. The plasma concentrations of L-arginine-related biomarkers, melatonin, and cortisol were measured every 3 h during a 24-h period. In addition, 24-h blood pressure recordings were performed. In non-night shift workers, L-arginine and homoarginine plasma concentrations showed diurnal variation with a 12-h period, which were both attenuated in night shift workers. ADMA and SDMA showed a 24-h rhythmicity with no significant differences in phase between night shift and non-night shift workers. The plasma profiles of melatonin and cortisol were not significantly different between both groups, suggesting that the rotating night shift work does not have a major influence on central suprachiasmatic nuclei clock timing. In addition, systolic and diastolic blood pressure patterns were similar between both groups. Our data show diurnal variation of dimethylarginines with the timing of their acrophases corresponding to the published timing of the peak incidence of cardiac ischemic events.

## 1. Introduction

Circadian rhythms underlie many physiological functions of our body [1]. The main regulatory element is the central circadian clock located in the suprachiasmatic nuclei (SCN) [2]. It is strongly influenced by light and other environmental triggers and is believed to signal through synthesis of melatonin [3] and cortisol. The production of melatonin is driven directly by the SCN clock and is acutely inhibited by light. Cortisol is another hormone with a prominent circadian rhythm, driven by the SCN and the autonomic system, with cortisol levels rising steeply in the early morning hours and levelling off in the course of the day [4].

Circadian rhythms are also important regulators of various functions of the cardiovascular system that go far beyond the widest and longest known function, i.e., the day-night variability of blood pressure [5]. More recent studies showed that endothelium-dependent, nitric oxide (NO)-mediated vascular function and arterial stiffness underlie distinct diurnal changes [6]. The nighttime dip in blood pressure is a physiological function that is not primarily based on the time of day, but on the level of activity, as evidenced by the differences in blood pressure patterns between (day-active) humans and (night-active) rodents [7]. Absence of the nighttime dip is associated with an elevated risk of cardiovascular disease [8,9]; it is often observed in individuals with disrupted circadian rhythms such as night shift workers [10]. Local circadian clocks that exist in both cardiomyocytes [11] and cells from vascular tissues [12] have been shown to act by cyclic translation of clock genes such as BMAL1 and PER2, their activities governing circadian control of myocardial and vascular function in concert with the central SCN circadian clock. Importantly, diverse aspects of vascular function, such as the endothelial regulation of vascular tone, are also controlled by circadian oscillators [13]. From many clinical studies, the onset of myocardial infarction has been known to peak in the morning hours [14], as does the time of onset of sudden cardiac death [15].

Methylated analogues of the amino acid L-arginine, a substrate of endothelial NO synthase, have gained major importance as prognostic biomarkers for cardiovascular disease and mortality [16,17]. Asymmetric dimethylarginine (ADMA) acts as a directly competitive inhibitor of NO synthase activity, its concentration being linked to vascular endothelial dysfunction [18]. By contrast, symmetric dimethylarginine (SDMA) does not directly interfere with NO synthase substrate binding, but—like ADMA–competitively inhibits L-arginine uptake into cells via the y^+^ system of transporters for cationic amino acids [19]. SDMA is a marker of risk after ischemic stroke [20,21]. Homoarginine is another methylated L-arginine analogue, deficiency of which has been associated with stroke and post-stroke outcome as well as with congestive heart failure [22,23].

We investigated whether the plasma concentrations of L-arginine and its methylated analogues that influence endothelial NO generation, ADMA, SDMA, and homoarginine, display diurnal variation, and whether this variation parallels the diurnal variation in blood pressure. We compared healthy adults with apparently healthy, long-term night shift workers to evaluate the effect of circadian misalignment and mistimed sleep and feeding on the daily rhythms. These may have causative influences on the development of vascular dysfunction and cardiovascular disease, as murine models with genetically modified CLOCK genes display endothelial dysfunction and cardiometabolic disease phenotypes.

## 2. Materials and Methods

### 2.1. Study Participants

Sixty apparently healthy individuals undergoing rotating night shift work and 12 apparently healthy individuals who had never undertaken night shift work were recruited for this study. Inclusion criteria comprised rotating night shift work (≥three night shifts per month) for a duration of at least 6 months. Exclusion criteria comprised any severe somatic or psychiatric disease, long-distance flights (3 time zones or more) within 4 weeks before the start of the study, intake of melatonin supplements within 4 weeks before commencing the study, regular caffeine intake of more than 750 mg/day, and blood donation within 60 days before inclusion in the study. All study participants gave their written informed consent to participate in the study. The study protocol was approved by the Ethics Committee of the Chamber of Physicians of Hamburg (decision nos. PV4287 and PVN4419).

### 2.2. Study Protocol

All study participants underwent an initial study visit comprising medical history, clinical examination, and blood sampling for routine laboratory tests. The participants had their blood pressure (BP) measured continuously for 24 h. Starting the next morning at 07:00 h, all study participants were confined to an in-patient ward for a total of 26 h. Room lights (neon tubes, 280 lux) were switched off from 21:00 h to 07:00 h, and standardized meals were provided at 07:30 h (breakfast), 12:00 h (lunch), and 19:00 h (dinner). All study participants were encouraged to sleep when the lights were switched off, but this was not enforced. During the entire in-patient period, blood samples were drawn through an in-dwelling cannula in an antecubital vein at 3-h intervals, starting at 08:00 h, for the determination of plasma biomarkers, melatonin, and cortisol. Plasma samples were immediately placed on ice, centrifuged within 30 min of blood withdrawal (4 °C, 1620× *g*, 10 min), and plasma aliquots were stored frozen at −80 °C until analysis.

Night shift workers had a broad variety of professional activities, ranging from health care workers to police officers, firemen, and taxi and bus drivers. Night shift workers were studied within 3–7 days after the end of a regular night shift; their night shift schedules were very variable, with the majority of study participants undertaking rotating night shift work. Non-night shift workers had various employments that exclusively involved daytime work.

### 2.3. Measurement of L-Arginine and Its Metabolites by Liquid Chromatography–Tandem Mass Spectrometry

L-Arginine, ADMA, SDMA, and homoarginine were determined by liquid chromatography–tandem mass spectrometry (LC–MS/MS) using a previously validated method [24]. In brief, 25 µL aliquots of plasma were diluted with 100 µL of stable isotope labeled L-arginine, ADMA, SDMA, and homoarginine dissolved in methanol and proteins were precipitated with methanol. After protein precipitation, analytes were converted to their butyl ester derivatives and analyzed by LC–MS/MS (Varian 1200, Agilent Technologies, Santa Clara, CA, USA). Separation of analytes from major matrix components was achieved with a Polaris C18-Ether column (Varian; 50 × 2.0 mm^2^) using an elution gradient of (A) 0.1% formic acid in water and (B) acetonitrile/methanol (50/50, *v*/*v*) containing 0.1% aqueous formic acid (95/5, A/B (*v*/*v*) to 50/50 over 2 min) at 30 °C, with a flow rate of 0.3 mL/min. Analyte concentrations were calculated using calibration curves based on four concentration levels of each analyte. Quality controls (QC) were run in two concentrations in triplicate. The second analysis was done on the QC samples to assess coefficient of variation and bias of QC, which was below 15% for all analytes. All other laboratory values were measured using routine clinical laboratory methods.

### 2.4. Assessment of Plasma Melatonin and Cortisol

Melatonin and cortisol concentrations were measured in lithium heparin plasma samples by specific radioimmunoassays with reagents obtained from Stockgrand Ltd., Norwich, UK (University of Surrey) as described previously [25,26]. All samples from one participant were run in the same assay and measured in duplicate. Limit of detection of the melatonin and cortisol assays (mean ± SD) was 2.8 ± 1.0 pg/mL and 1.6 ± 0.7 nmol/L, respectively. For both assays, the interassay coefficients of variation (CVs) of low, medium, high, and very high QCs were <12% (range 5.4–11.4%; N = 30 and 33, respectively, for melatonin and cortisol).

### 2.5. 24-h Blood Pressure Measurements

Using the BPLab^®^ ambulatory blood pressure monitoring system (OOO Petr Telegin, Nizhny Novgorod, Russia), 24-h blood pressure measurements were performed. Measurements were performed every 15 min during daytime (07:00 h to 21:00 h), and every 30 min during nighttime (21:00 h to 07:00 h).

### 2.6. Statistical Analyses

Mean 24-h biomarker concentrations in plasma were calculated to compare shift workers and non-shift workers. Differences between the two groups were tested for significance by two-tailed *t* test. Cosinor analysis was performed on all biomarker data using the CosinorOnline application (https://cosinor.online/app/cosinor.php [27], accessed on 18 August 2023). Cosinor analysis is based upon fitting a cosinor curve to the data and assessing the curve that results in the least squared deviations of the measured data from the fitted curve [28]. All data were tested for period lengths of 12 h and 24 h; the better fit was used in the subsequent analysis. By comparing the size of the amplitude in a data set with the least squares of the deviation of the measured data from the fitted curve, the zero amplitude test calculates the probability of the presence of a biological rhythm with the give period length. Therefore, the zero amplitude test was performed to test for the presence of a significant diurnal variation within each subject’s data set. Hourly mean blood pressure was tested for significant differences by two-way ANOVA with Bonferroni correction for multiple comparisons; individual missing blood pressure values were substituted according to the carry-forward principle. Dipper status and re-classification scores were assessed using the chi-square test. P for trend is given for comparisons of more than two groups; Fisher’s exact test was used for calculating statistical significances for the comparison of categorical variables from two groups. Data are presented as mean ± standard deviation (SD); 24-h time profiles were plotted as mean ± standard error of the mean (SEM) to improve graphic visibility of differences between group means, as indicated in the Figure legends. A level of statistical significance of *p* < 0.05 was applied.

## 3. Results

### 3.1. Baseline Characteristics

The night shift workers had a mean age of 35.8 ± 11.7 years (mean ± SD), as compared to 37.1 ± 11.6 years for the non-night shift workers. Approximately half of the night shift workers (*n* = 35, 58%) and non-night shift workers (*n* = 6, 50%) were females. Night shift workers had a work history of 10.8 ± 9.4 years of night shift with a mean 7.9 ± 4.4 night shifts per month, whilst none of the non-night shift workers had ever worked in night shifts. Mean systolic and diastolic blood pressure was 122.1 ± 10.9/73.6 ± 8.7 mm Hg in night shift workers and 116.8 ± 13.9/74.4 ± 9.2 mm Hg in non-night shift workers (*p* > 0.05 between both groups). Body mass index (BMI) tended to be higher in night shift workers than in non-night shift workers, but this difference did not achieve statistical significance (27.1 ± 6.0 vs. 23.8 ± 2.7 kg/m^2^, respectively; *p* = 0.07). Further baseline characteristics of both groups are shown in Table 1.

### 3.2. Diurnal Variation of L-Arginine and Its Metabolites

**Figure 1:**
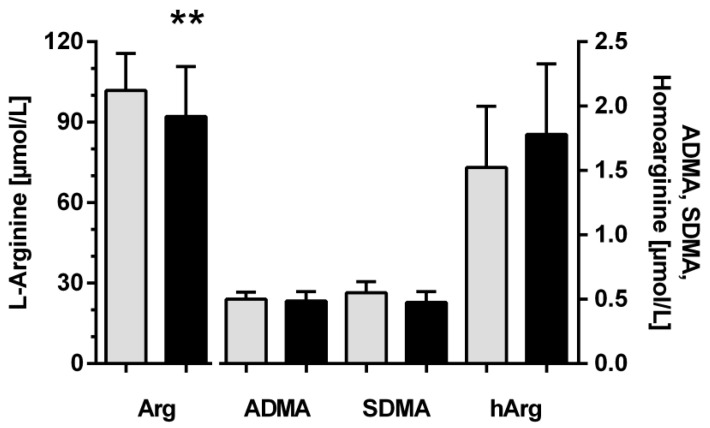
24-h means of the plasma concentrations of L-arginine, ADMA, SDMA, and homoarginine in non-night shift workers (grey bars; *n* = 12) and night shift workers (black bars; *n* = 60). Data are mean ± standard deviation. ** *p* < 0.01 vs. non-night shift workers.

The overall mean L-arginine plasma concentration was slightly, but significantly, lower in night shift workers than in non-night shift workers (Figure 1). For ADMA, SDMA, and homoarginine, there were no significant differences between both groups.

**Figure 2:**
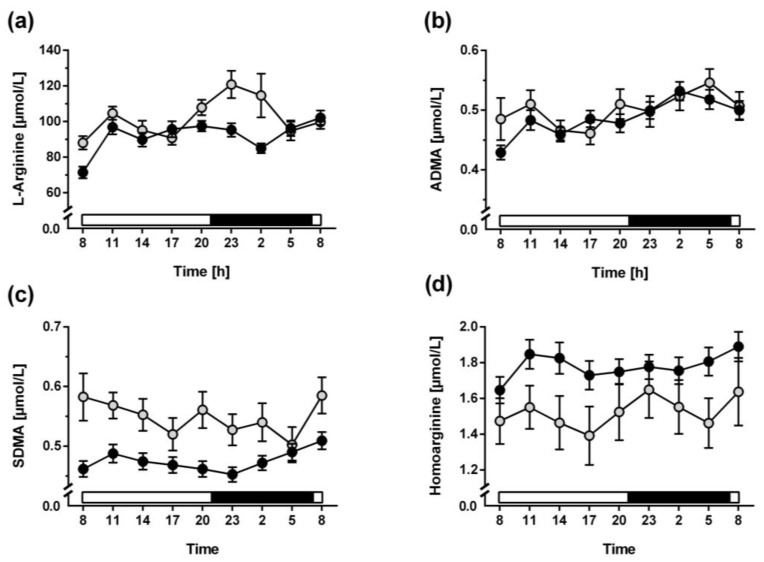
Plasma concentration profiles of L-arginine (**a**), ADMA (**b**), SDMA (**c**), and homoarginine (**d**) from 3-hourly blood samples during one full 24-h period. Data are mean ± standard error of the mean. The bar at the bottom of each graph indicates the timing of the light (open bar) and dark time periods (filled bar). Open circles represent data from non-night shift workers, filled circles represent data from night shift workers.

**Figure 3:**
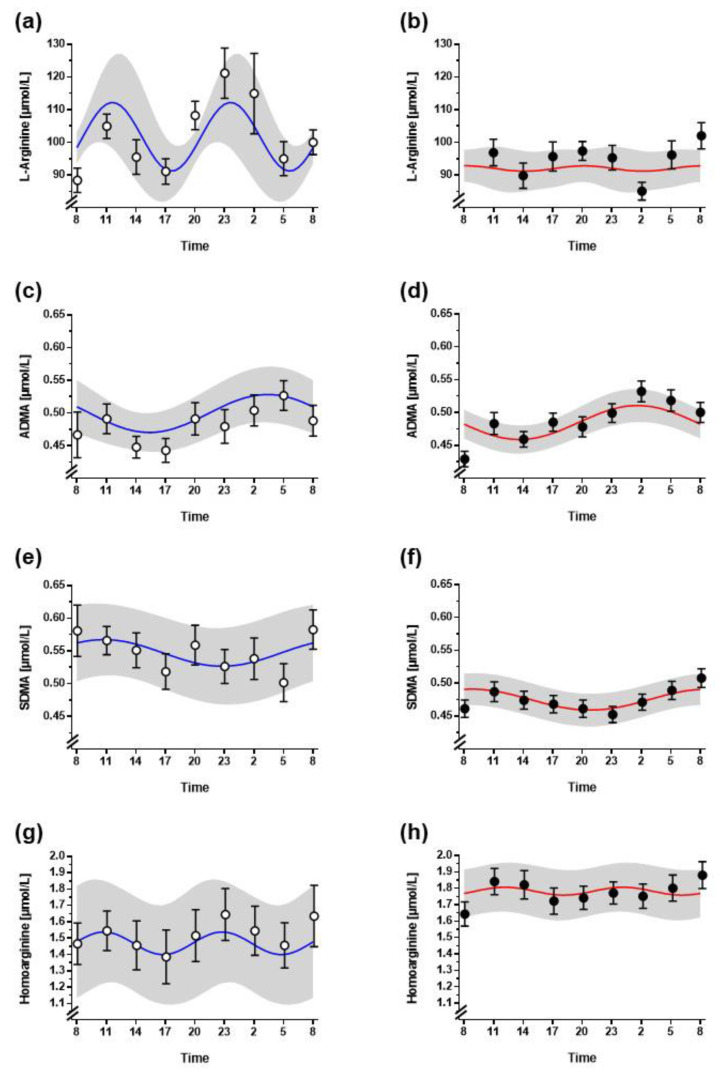
Cosinor analysis of daily profiles of L-arginine (**a**,**b**), ADMA (**c**,**d**), SDMA (**e**,**f**), and homoarginine (**g**,**h**) with mean optimized cosinor curves plotted over means ± standard error of the mean of original measurements. The shaded area represents the 95% confidence interval for the cosinor curves. Graphs in the left column represent data from non-night shift workers (open circles, blue cosinor lines), graphs in the right column represent data from night shift workers (filled circles, red cosinor lines).

There was no significant diurnal variation of L-arginine in non-night shift workers, even though L arginine plasma concentration was higher in the morning and in the evening. However, high intra- and inter-individual variation of the measured L-arginine concentrations resulted in a non-significant zero-amplitude test. Whilst variability of L-arginine concentrations was attenuated in night shift workers, so was the amplitude (Figure 2a). Cosinor analysis was applied to test whether a 12-h or 24-h rhythm could be significantly fitted to the data; this analysis revealed that L-arginine concentrations were best fitted to a cosinor curve with a period of 12 h and an amplitude of 14.2 ± 10.2 µmol/L in non-night shift workers (Figure 3a; Table 2). Diurnal variability was almost completely absent in night shift workers (Figure 3b).

A significant diurnal variation of plasma ADMA concentration was observed in both night shift workers and non-night shift workers (Figure 2b). In cosinor analysis, the best fitted period was 24 h with the acrophase occurring in the early morning hours (03:22 h in non-night shift workers and 03:10 h in night shift workers (Figure 3c,d, Table 2)). For plasma SDMA concentration, no significant diurnal variation was observed (Figure 2c and Figure 3e,f). This was largely due to high variation in individual SDMA concentrations. Homoarginine showed no significant diurnal variation in non-night shift workers; it varied with a period of 12 h but a very small amplitude of 0.09 µmol/L (Figure 2d and Figure 3g), which had a phase very similar to that of L-arginine, but a high inter-individual variability of the measured plasma concentrations. In night shift workers this rhythm showed less inter-individual variability; it was significantly fitted to a 12-h period with an amplitude of 0.16 µmol/L (Figure 3h).

### 3.3. Hormone Markers of Circadian Timing

**Figure 4:**
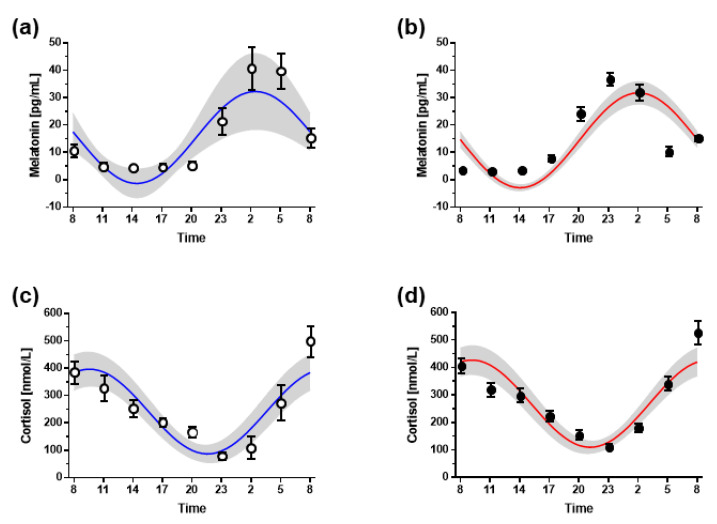
Cosinor analysis of daily profiles of plasma melatonin (**a**,**b**) and cortisol (**c**,**d**) concentrations with mean optimized cosinor curves plotted over means ± standard error of the mean of original measurements. The shaded area represents the 95% confidence interval for the cosinor curves. Graphs in the left column represent data from non-night shift workers (open circles, blue cosinor lines), graphs in the right column represent data from night shift workers (filled circles, red cosinor lines). Open circles represent data from non-night shift workers, filled circles represent data from night shift workers.

Plasma melatonin concentrations showed a steep increase with the onset of darkness (lights were turned off at 21:00 h); its concentration peaked between 02:00 h and 03:00 h, and declined sharply in the early morning hours. This pattern was almost identical in both groups (Figure 4a,b). Cosinor analysis revealed a non-significant about 1-h phase advance of the circadian rhythm of plasma melatonin in night shift workers, with the acrophase determined at 02:27 ± 3:07 h in night shift workers and at 02:53 ± 1:43 h in non-night shift workers (Table 3).

Plasma cortisol concentrations sharply increased during the early morning hours, i.e., between 02:00 h and 08:00 h, and declined continuously during the daytime hours (Figure 4c,d). Similar to melatonin, this pattern was almost identical in both groups, with the cortisol acrophase determined at 09:41 ± 1:40 h in non-night shift workers and at 09:09 ± 2:20 h in night shift workers (Table 3).

### 3.4. 24-h Profiles of Blood Pressure

**Figure 5:**
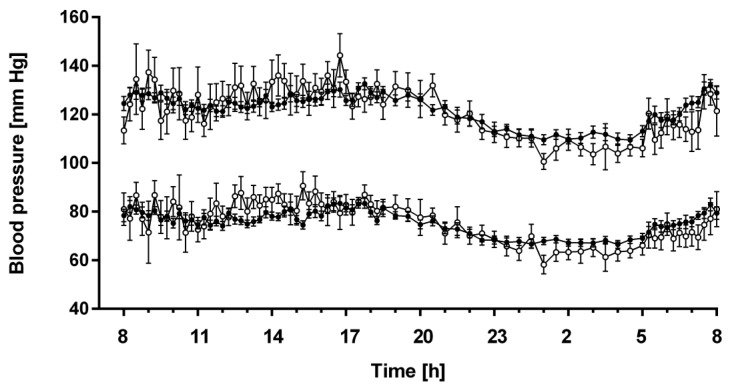
24-h profiles of systolic and diastolic blood pressure in non-night shift workers (open circles) and night shift workers (filled circles). Data are given as mean ± standard error of the mean from measurements taken at 15 min intervals from 07:00 h to 21:00 h and at 30 min intervals from 21:00 h to 07:00 h.

Blood pressure profiles were not significantly different between night shift workers and non-night shift workers and showed similar physiological day-night differences in both groups (Figure 5). There was a slight trend towards more pronounced day-night differences in non-night shift workers, with slightly higher daytime and slightly lower night-time blood pressure values as compared to night shift workers. This trend, however, showed no statistical significance, neither for systolic nor for diastolic blood pressure. The numeric mean drop in systolic and diastolic blood pressure during nighttime was 9.3 ± 9.4 mm Hg and 7.3 ± 7.2 mm Hg in night shift workers, respectively, and 8.0 ± 10.6 mm Hg and 7.9 ± 6.9 mm Hg in non-night shift workers, respectively. Of the night shift workers, 34.5% were dippers as opposed to 45.5% of the non-night shift workers (*p* > 0.05 between both groups).

## 4. Discussion

The present study has three major findings: Firstly, the plasma concentration of L-arginine shows diurnal variability with a high variation in inter-individual data that resembles to a non-significant 12-hourly rhythm; however, in night shift workers, diminished inter-individual variation of the data and diminished amplitude result in a flattened diurnal profile of plasma L-arginine. Secondly, the methylated L-arginine derivatives, ADMA and SDMA, show daily rhythms with a peak in the early morning hours. Finally, homoarginine displays a 12-hourly rhythm which is significantly attenuated in night shift workers. These results strongly suggest differential regulatory mechanisms driving the diurnal variation in plasma levels of the L-arginine-related metabolites.

L-Arginine is a semi-essential amino acid that is quite ubiquitously present in cells and tissues. We consume a high amount of L-arginine with our daily food; the Western diet has been calculated to contain a mean 4–5 g L-arginine/day [29,30]). The physiological plasma concentration of this amino acid has been reported as 60–130 µmol/L [31] and 42–130 µmol/L [32]. We have more recently confirmed these plasma concentration ranges using state-of-the art methodology of LC–MS/MS with a reference range of 41–114 µmol/L in the Framingham Offspring Study [33]. The same analytical technology was applied in the present study. The plasma concentrations measured in the present study fall well into this reference range, both in non-night shift workers and night shift workers. The major difference that we observed was that of a clearly visible 12-hourly rhythm with two peaks of plasma L-arginine in the morning and in the late evening hours in non-night shift workers. This rhythm was almost completely diminished in night shift workers, resulting in a significantly lower 24-h mean plasma concentration. Night shift work is associated with irregular meal times and higher consumption of less healthy (fast) food during night shifts [34,35,36,37]. Thus, irregular timing of dietary intake of L-arginine may explain our present observation.

The two methylated L-arginine metabolites that have been shown to inhibit NO synthesis by different mechanisms, ADMA and SDMA; both showed a daily rhythm in our study. ADMA plasma concentrations peaked in the early morning hours, whilst SDMA concentrations peaked in the late morning hours. The rhythm was significant in cosinor zero-amplitude tests for ADMA in both groups of study participants, but it failed to reach formal statistical significance for SDMA. ADMA is a direct, competitive inhibitor of NO synthesis, where it displaces L-arginine from the substrate-binding pocket. By this mechanism, ADMA causes endothelial dysfunction, i.e., the inability of a blood vessel to dilate in response to physiological or pharmacological stimuli of endothelial NO release [18]. Endothelium-dependent, NO-mediated vasodilation has been shown to follow a diurnal rhythm in healthy males that reaches its nadir when the diurnal ADMA profile reaches its acrophase, i.e., in the early morning hours [38].

ADMA is also used clinically as a risk biomarker of total mortality and cardiovascular event rate [16]. A diurnal rhythm of ADMA with its acrophase in the morning hours has been observed by Bergheanu and co-workers in patients admitted to hospital for emergency coronary revascularization due to acute myocardial infarction [39]. The present study extends this observation to show that apparently healthy adults display the same time-of-day variation of plasma ADMA. Taken together, these findings are suggestive of a pathophysiological role of ADMA-induced inhibition of endothelial NO production, vascular dysfunction and enhanced platelet activity [40], and timing of myocardial infarction in the early morning hours. Peaks in the rates of excretion of urinary nitrate (an oxidative metabolite of NO) and cyclic guanosine monophosphate (cGMP, the second messenger of NO) were reported to concurrently occur between 17:00 h and 20:00 h [41], whereas Zhadanova et al. [42] observed the peak in plasma cGMP during the night. Urinary nitrate, however, is a rather unspecific, indirect measure of NO formation, and cGMP is also generated by stimuli other than NO.

The plasma concentration of ADMA is most strongly influenced by the activity of dimethylarginine dimethylaminohydrolase (DDAH), its major metabolising enzyme [43]. DDAH is expressed in various tissues in two distinct isoforms, DDAH1 and DDAH2 [44]. Of these, gene silencing studies in rodents have shown that DDAH1, but not DDAH2, modulates ADMA plasma concentrations [45]. We have confirmed this experimental finding in a genome-wide association study in humans, in which the DDAH1 gene emerged as the single major gene significantly associated with ADMA [46]. By contrast, SDMA is not a substrate of DDAH, but it is enzymatically cleaved by alanine glyoxylate aminotransferase (AGXT)2. This finding also emerged from a genome-wide association study that proved the AGXT2 gene as the only one significantly associated with SDMA [46]. Indeed, we could confirm experimentally in the same study that AGXT2 metabolizes SDMA [46]. Our present finding that the daily rhythms of ADMA and SDMA have different phase timing is supportive of the different metabolic cleavage pathways that determine their plasma concentrations.

Homoarginine is an endogenous, non-proteinogenic amino acid that is formed endogenously from L-arginine and L-lysine by the activity of L-arginine–glycine aminotransferase (AGAT) [23]. This enzyme also catalyses the conversion of L-arginine and L-glycine to guanidinoacetate and L-ornithine, which is the rate-limiting step in creatine synthesis and regarded as the principal enzymatic activity of this enzyme [47]. Thus, homoarginine is an alternative product of AGAT. Nonetheless, low homoarginine has been shown to be associated with stroke size, mortality, and congestive heart failure [23,48,49]. Homoarginine, but not creatine supplementation, has been shown to ameliorate stroke size and improve cardiac contractile function in AGAT knockout mice [23,50]. Recently, we have defined the sex-specific 2.5th and 97.5th percentiles of homoarginine plasma concentrations to be 0.84 and 3.89 μmol/L in women and 0.98 and 4.10 μmol/L in men, respectively [51]. The homoarginine plasma concentrations measured in the present study are within these reference ranges quantified using the same analytical platform. Nevertheless, homoarginine plasma concentrations showed a diurnal variation with a 12-h period, which was significantly attenuated in night shift workers, indicating that both, endogenous and exogenous sources for circulating homoarginine are likely. The only well-characterised dietary source of homoarginine is grass pea (lathyrus sativus), a legume that has toxic effects when consumed for prolonged periods of time and at higher doses. Grass pea seeds are not a usual part of the diet in central Europe; therefore, it remains unclear if the 12-hourly rhythm of plasma homoarginine observed in the present study is the result of another, yet unknown dietary component or an endogenous metabolic rhythm.

The endogenous circadian rhythms of melatonin and cortisol showed no differences in phase or amplitude between non-night shift workers and night shift workers in this study. The timing of acrophase and nadir of both hormones were consistent with reported data from the literature in both groups [25]. Therefore, we cannot verify a major disruption of the SCN-driven circadian rhythms in the rotating night shift workers that we included in this study. This finding, made during normal entrained conditions, confirms previous observations made in the setting of constant routines and simulated night and day shifts [52]. The switching off of room lights at 21:00 h may explain the sudden rise in melatonin after 21:00 h since the light may have suppressed any melatonin onset before that. Moreover, at 07:00 h, lights may have reinforced the last part of the natural melatonin decline.

We also did not find a major change in the diurnal variation of systolic and diastolic blood pressure between night shift workers and non-night shift workers. With the exception of a slight dampening of the day–night difference in blood pressure in the night shift workers, blood pressure profiles looked very similar in both groups. We did not find any interrelationships suggestive of physiological significance between the diurnal profiles of L-arginine and the methylated L-arginine metabolites and that of blood pressure. The acrophase of ADMA, which one would expect to increase blood pressure due to inhibition of nitric oxide synthesis, occurred at the time when blood pressure was lowest. In addition, the biphasic diurnal profile of L-arginine was not synchronous with blood pressure variability. SDMA is known to have a comparatively indirect influence on vascular tone and, thus, blood pressure; and the role of homoarginine as a potential substrate of NO synthase has remained unclear.

Our study is limited by the fact that, due to legal restrictions, rotating shift workers that we were able to recruit performed a rather limited number of night shifts per month. Although we had found a significant effect of time-of-day-adjusted light supplementation on diurnal blood pressure patterns in a recent study in a similar group of rotating shift workers, we did not find a significant deviation of melatonin and cortisol profiles from healthy controls either [10]. As the participants were studied within a time window of 3–7 days after their most recent night shift, the effects that we report here relate to medium-to-long-term effects of night shift work rather than to acute effects. We therefore cannot extrapolate our present findings to predict whether a single night shift might acutely exert similar effects on L-arginine and its metabolites. Finally, although the environmental conditions of the study day were kept as constant as possible, our study was not performed in an enclosed facility under exclusion of external stimuli.

## 5. Conclusions

We report here a biphasic diurnal variation of L-arginine and homoarginine plasma concentrations, which were both diminished in night shift workers. These data support a strong dietary influence on L-arginine plasma concentrations, whilst the underlying cause of the time of day variation in homoarginine remains elusive. The direct NO synthesis-inhibitor, ADMA, showed diurnal variation, the phase of which was quite synchronous with the reported timing of onset of myocardial infarction. This finding is suggestive of a pathophysiological role of ADMA-induced inhibition of nitric oxide-mediated vasodilation in the onset of myocardial infarction; it supports the relevance of ADMA as a cardiovascular risk biomarker.

## Figures and Tables

**Table 1:** Baseline characteristics of the study participants.

	Night Shift Workers	Non-Night Shift Workers	*p*
**Demographics**			
No. of individuals	60	12	n.a.
Age (years)	35.8 ± 11.7	37.1 ± 11.6	n.s.
Sex (f/m)	35/25	6/6	n.s.
**Anthropometrics**			
Height (cm)	173.7 ± 8.5	176.3 ± 9.0	n.s.
Weight (kg)	81.6 ± 19.5	74.7 ± 14.6	n.s.
BMI (kg/m^2^)	27.1 ± 6.0	23.8 ± 2.7	0.07
Systolic blood pressure (mm Hg)	122.1 ± 10.9	116.8 ± 13.9	n.s.
Diastolic blood pressure (mm Hg)	73.6 ± 8.7	74.4 ± 9.2	n.s.
Heart Rate (1/min)	64.8 ± 11.5	68.6 ± 12.5	n.s.
**Work History**			
Night shifts since (years)	10.8 ± 9.4	-	n.a.
No. of night shifts/month	7.9 ± 4.4	-	n.a.
**Laboratory Parameters**			
Glucose (mg/dL)	88.5 ± 10.5	86.6 ± 5.9	n.s.
HbA1c (%)	5.1 ± 0.4	5.0 ± 0.3	n.s.
Total cholesterol (mg/dL)	179.7 ± 36.3	166.9 ± 33.2	n.s.
Triglycerides (mg/dL)	105.2 ± 46.0	86.3 ± 34.3	n.s.
LDL cholesterol (mg/dL)	99.8 ± 33.0	84.4 ± 33.2	n.s.
HDL cholesterol (mg/dL)	57.2 ± 17.9	65.2 ± 19.1	n.s.
Creatinine (mg/dL)	0.83 ± 0.16	0.75 ± 0.16	n.s.

Continuous variables are presented as mean ± standard deviation (SD); categorical variables are shown as absolute numbers. Abbreviations: HbA1c, glycosylated hemoglobin; HDL, high density lipoprotein; LDL, low density lipoprotein; n.a., not assessed; n.s., not significant (*p* values below 0.1 are specified in this Table).

**Table 2:** Diurnal rhythm characteristics of L-arginine metabolites.

	L-Arginine	ADMA	SDMA	Homoarginine
**Non-night shift workers**				
Period (h)	12 h	24 h	24 h	12 h
Mesor [µmol/L]	101.7 ± 14.9	0.50 ± 0.05	0.55 ± 0.09	1.47 ± 0.47
Acrophase *	n.d.	04:05 ± 2:03	n.d.	n.d.
Nadir *	n.d.	15:42 ± 2:07	n.d.	n.d.
Amplitude [µmol/L]	14.2 ± 10.2	0.04 ± 0.02	0.03 ± 0.02	0.18 ± 0.12
Zero-amplitude test (*p* value)	0.157	0.038	0.366	0.077
**Night shift workers**				
Period (h)	12 h	24 h	24 h	12 h
Mesor [µmol/L]	92.0 ± 18.9	0.49 ± 0.08	0.47 ± 0.08	1.76 ± 0.59
Acrophase *	n.d.	03:10 ± 1:04	n.d.	00:06 ± 3:42 12:06 ± 3:42
Nadir *	n.d.	14:40 ± 1:06	n.d.	06:06 ± 3:42 18:06 ± 3:42
Amplitude [µmol/L]	11.9 ± 11.3	0.06 ± 0.04	0.04 ± 0.03	0.16 ± 0.12
Zero-amplitude test (*p* value)	0.986	0.009	0.150	0.026

Data are presented as mean ± standard deviation (SD) where appropriate. Abbreviations: ADMA, asymmetric dimethylarginine; SDMA, symmetric dimethylarginine. * Acrophase and nadir are given as clock times (h:min) in the 24 h format. These times were not calculated in the absence of a significant diurnal variation (n.d., not determined).

**Table 3:** Rhythm characteristics of melatonin and cortisol.

	Melatonin	Cortisol
**Non-night shift workers**		
Period (h)	24 h	24 h
Mesor [µmol/L]	15.47 ± 8.97	241.9 ± 54.8
Acrophase *	02:53 ± 1:43	09:41 ± 1:40
Nadir *	14:21 ± 2:55	21:41 ± 1.40
Amplitude [µmol/L]	18.14 ± 1.41	166.5 ± 56.9
Zero-amplitude test (*p* value)	0.008	0.007
**Night shift workers**		
Period (h)	24 h	24 h
Mesor [µmol/L]	13.24 ± 7.34	265.0 ± 117.6
Acrophase *	02:27 ± 3:06	09:09 ± 2:20
Nadir *	13:35 ± 2:07	21:09 ± 3:08
Amplitude [µmol/L]	17.10 ± 10.85	183.0 ± 92.3
Zero-amplitude test (*p* value)	0.005	0.005

* Acrophase and nadir are given as clock times (h:min) in the 24 h format.

## Data Availability

The data presented in this study are available on request from the corresponding author. The data are not publicly available due to privacy reasons imposed by local Data Protection Laws.
